# Prognostic Value of Ki-67 in Patients With Resected Triple-Negative Breast Cancer: A Meta-Analysis

**DOI:** 10.3389/fonc.2019.01068

**Published:** 2019-10-17

**Authors:** Qiang Wu, Guangzhi Ma, Yunfu Deng, Wuxia Luo, Yaqin Zhao, Wen Li, Qinghua Zhou

**Affiliations:** ^1^Lung Cancer Center & Institute, West China Hospital, Sichuan University, Chengdu, China; ^2^Department of Oncology, Chengdu First People's Hospital, Chengdu, China; ^3^Cancer Center, West China Hospital, Sichuan University, Chengdu, China

**Keywords:** Ki-67, triple-negative breast cancer, TNBC, prognosis, meta-analysis

## Abstract

**Background:** Ki-67 is a widely used marker of tumor proliferation, but the prognostic value of ki-67 in triple-negative breast cancer (TNBC) has not been comprehensively reviewed. This meta-analysis was conducted to evaluate the association between ki-67 expression and survival of patients with resected TNBC.

**Materials and Methods:** Relevant studies, evaluating the prognostic impact of pretreatment ki-67 in resected TNBC patients, were identified from PubMed, Embase, Web of Science, China National Knowledge Infrastructure, and Cochrane Library until March 14, 2019. Hazard ratios (HRs) with 95% confidence intervals (CI) were calculated as effect values for disease-free survival (DFS) and overall survival (OS).

**Results:** In present meta-analysis, 35 studies with 7,716 enrolled patients were eligible for inclusion. Pooled results showed that a high ki-67 expression was significantly associated with poor DFS (HR = 1.73, 95% CI: 1.45–2.07, *p* < 0.001) and poor OS (HR = 1.65, 95% CI: 1.27–2.14, *p* < 0.001) in resected TNBC. In the subgroup analysis, when a cutoff of Ki-67 staining ≥40% was applied, the pooled HR for DFS and OS was 2.30 (95% CI 1.54–3.44, *p* < 0.001) and 2.95 (95% CI 1.67–5.19, *p* < 0.001), respectively.

**Conclusion:** A high Ki-67 expression is a poor prognostic factor of resected TNBC. The cut-off of ki-67 ≥40% is associated with a greater risk of recurrence and death compared with lower expression rates, despite the Ki-67 threshold with the greatest prognostic significance is as yet unknown.

## Introduction

Breast cancer is one of the most frequently diagnosed cancers and the leading cause of cancer morbidity in women worldwide. It affected more than 1.6 million individuals in 2012 and constituted ~15% of all cancer-related deaths among females (1). Triple-negative breast cancer (TNBC) is a subtype of breast cancer and accounts for about 12 to 17% of all breast cancers (2). Due to lacking the expression of estrogen receptor (ER), progesterone receptor (PR), and human epidermal growth factor receptor type 2 (HER2) in tumor cells, patients with TNBC are neither sensitive to endocrine therapy nor therapies targeted to HER2 (3). TNBC is usually a high-grade invasive ductal carcinoma without a special pathological type, and it is also a heterogeneous disease because some of these patients are obviously sensitive to chemotherapy with likelihood to achieve a favorable prognosis (4, 5). Thus, sufficient and valid prognostic factors of TNBC should be identified.

Ki-67, a non-histone nuclear protein, is present in the cell nucleus during all of the active phases of the cell cycle (G_1_, S, G_2_, and mitosis) but absent in quiescent cells (G_0_), which makes it a widely used biomarker of tumor proliferation and a crucial element of pathological assessment (6, 7). The prognostic significance of Ki-67 has been extensively evaluated in various malignancies, including breast cancer. Ki-67 is established as a vital factor in the distinction between luminal A and luminal B breast cancer subtypes by the 2011 and 2013 St. Gallen International Breast Cancer Conference (8, 9). Unlike its role in luminal diseases whose low Ki-67 expression achieves an enhanced prognosis after standard systematic treatments, the prognostic value of Ki-67 in TNBC is still unclear and no consensus has been reached (10). Therefore, this study focused on the assessment of the prognostic value of Ki-67 in resected TNBC patients.

## Methods

Our meta-analysis was conducted in line with the “Preferred Reporting Items for Systematic Reviews and Meta-Analyses” (PRISMA) statement (11).

### Search Strategy

A comprehensive electronic search of PubMed, Embase, Web of Science, China National Knowledge Infrastructure, and Cochrane Library was conducted without language restriction to identify all relevant full-length studies on the prognostic role of Ki-67 in patients with TNBC. To retrieve data as much as possible, we expand the search scope by using the keywords as follow: (“Ki-67” or “mib-1” or “proliferative marker”) and (“breast cancer” or “breast tumor” or “breast carcinoma” or “breast neoplasm”). The beginning date was not limited, and the search was up to March 14, 2019. References cited in eligible studies were also searched manually to obtain additional pertinent articles.

### Study Selection

The inclusion criteria were as follows: (1) studies or subsets in studies investigating the association between Ki-67 and prognosis in resected TNBC who has received neo-adjuvant or adjuvant treatment; (2) studies have adequate data for calculation including the hazard ratio (HR) and its corresponding 95% confidence interval (CI), and (3) the threshold value of Ki-67 was determined by pretreatment biopsy specimen.

The exclusion criteria were as follows: (1) non-original research articles with limited data, such as reviews, letters, comments, conference abstracts, or case reports; (2) studies without adequate survival or recurrence data for further calculation; (3) studies involving metastatic diseases; (4) overlapping or duplicate data; and (5) studies with a sample size of <30 analyzable cases.

### Data Extraction and Quality Assessment

The following data was extracted: first author's name, year of publication, country, study design and sample size, demographic characteristics (e.g., age, gender, and geographical background), cut-off value of Ki-67 expression, percentage of positive lymph nodes, treatment, and the HR with 95% CI of disease-free survival (DFS) and overall survival (OS). Multivariate outcomes were preferred when multivariate and univariate analyses performed simultaneously.

Newcastle–Ottawa Scale (NOS) was used to examine the qualities of the included studies (12). This evaluation tool covered the selection, comparability, and clinical outcomes, and studies were considered to be of high quality when they scored 6 or more.

### Statistical Analysis

Prognostic outcomes, including DFS and OS, were the primary endpoints of this study. DFS was defined as the interval period from the date of operation to the first observation of recurrence or the last follow-up without evidence of recurrence. OS was defined as the time from the first diagnosis of primary breast cancer to the time of death from any cause. HRs with 95% CIs for prognostic outcomes were extracted for further calculation. For those that were indirectly given in publications, published data and figures from original papers were extracted to calculate the corresponding HRs by utilizing the methods described by Tierney et al. (13).

Cochrane's Q (*P* < 0.1 was considered significant) and Higgins's *I*^2^ (*I*^2^ > 50% was considered substantially heterogeneous) statistic tests were used to evaluate the heterogeneity among the eligible studies (14). A fixed-effect model would be preferred in the analyses to acquire precise results if the heterogeneities were insignificant. Otherwise, a random-effect model should be utilized (15). Subgroup analyses were also conducted to investigate the role of Ki-67 in specific populations and the potential source of heterogeneity. Publication bias was assessed with a funnel plot via Egger's and Begg's tests, and results were considered insignificant when *P* > 0.1 (16). Sensitivity analysis was performed to explore the influence of individual studies on the summarized results.

Kaplan–Meier curves were recognized by Engauge Digitizer 4.1 (free software downloaded for http://getdata-graph-digitizer.com/). All tests were two sided, and *P* < 0.05 indicated statistical significance. Data analyses were performed with Stata 12.0 (StataCorp LP, TX, USA).

## Results

### Selection of Studies

**Figure 1:**
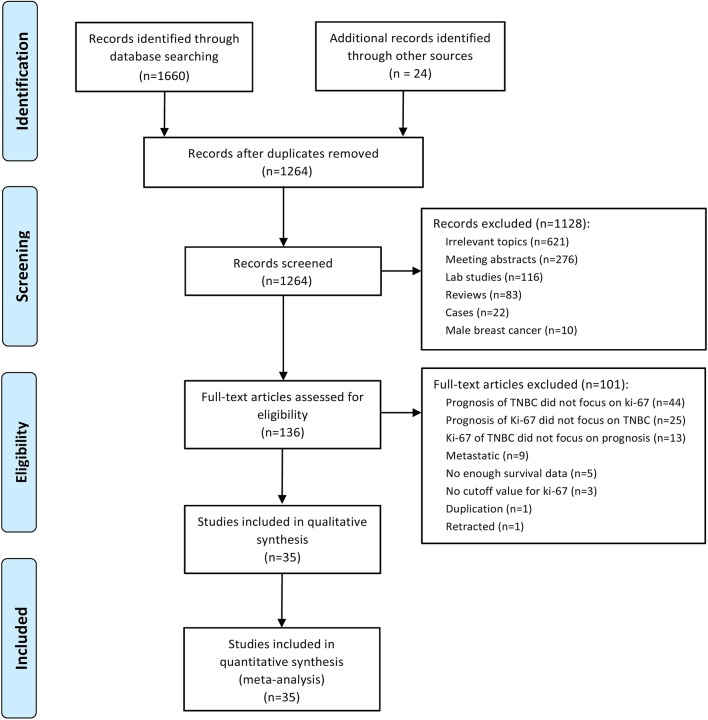
Flow chart of the included studies.

A total of 1,684 potential studies were identified by the search algorithm. After duplicates were removed, abstracts of the 1,264 remaining studies were reviewed. Of these studies, 1,128 were excluded, and 136 potentially relevant studies were selected for further examination. A total of 101 studies were excluded because the prognosis of TNBC did not focus on Ki-67 (*n* = 44); the prognosis of Ki-67 did not highlight TNBC (*n* = 25); and Ki-67 of TNBC did not cover prognosis (*n* = 13), metastatic disease (*n* = 9), insufficient survival data (*n* = 5), no cutoff for Ki-67 (*n* = 3), duplication (*n* = 1), and retracted study (*n* = 1). Finally, 35 studies regarding the prognostic role of Ki-67 in TNBC subjected to neo-adjuvant or adjuvant chemotherapy were eligible for this meta-analysis (17–51). The flow diagram of studies selection was summarized in Figure 1.

### Study Characteristics

A total of 7,716 patients with TNBC were enrolled in the 35 included studies for analyses. The patients' median age ranged from 50 to 60 years, and the median follow-up varied from 11 to 112 months. The cutoff of Ki-67 was 10%−50%. The article quality assessed by NOS was 6–9, and 80% of the included studies had a quality of 7–9. None of these studies included patients who underwent surgery alone without neoadjuvant or adjuvant treatment. Table 1 summarizes the main characteristics of the included studies.

**Table 1:** Characteristics of the included studies in this meta-analysis.

**References**	**Analysis of survival data**	**Neoadjuvant or adjuvant**	**Number of patients**	**Chemotherapy regimen**	**Region**	**Test tissue**	**Ki-67 cut off value (%)**	**ER/PR cut off value (%)**
Toyama et al. (17)	UVA	Adjuvant	71	5-FU	Asian	Operative specimen	10	10
Trere et al. (18)	Survival Curve	Adjuvant	24	CMF	European	Operative specimen	20	10
Lee et al. (19)	MVA	Adjuvant	1550	NR	Asian	Operative specimen	20	10
Nishimura et al. (20)	MVA	Adjuvant	356	CMF, CE(F), Taxane	Asian	Operative specimen	20	10
Wang et al. (21)	UVA	Adjuvant	42	Paclitaxel-based	Asian	Operative specimen	30	10
Keam et al. (23)	MVA	Neoadjuvant	105	Taxel/anthracyclin-based	Asian	Pre-NAC	10	10
Li et al. (24)	UVA	Adjuvant	125	NR	Asian	Operative specimen	NR	10
Masuda et al. (25)	MVA	Neoadjuvant	33	Taxel/anthracyclin-based	Asian	Pre-NAC	50	10
Miyashita et al. (26)	MVA	Adjuvant	102	NR	Asian	Operative specimen	40	1
Kashiwagi et al. (22)	Survival Curve	Adjuvant	190	5-FU/anthracyclin-based	Asian	Operative specimen	30	1
Munzone et al. (27)	Survival Curve	Adjuvant	496	Anthracyclin-based, CMF	European	Operative specimen	35	1
Ryu et al. (28)	UVA	Adjuvant	94	Anthracyclin-based	Asian	Operative specimen	10	10
Xue et al. (29)	MVA	Adjuvant	913	Taxel/anthracyclin-based, CMF	Asian	Operative specimen	14	5
Huang et al. (30)	MVA	Adjuvant	185	Taxel/anthracyclin-based, CMF	Asian	Operative specimen	20	1
Milde-Langosch et al. (31)	MVA	Adjuvant	95	CMF/EC	European	Operative specimen	NR	NR
Xu et al. (32)	MVA	Adjuvant	122	CMF	Asian	Operative specimen	10	10
Yamashita et al. (33)	MVA	Adjuvant	82	NR	Asian	Operative specimen	30	1
Zhang et al. (34)	MVA	Adjuvant	428	Taxel/anthracyclin-based	Asian	Operative specimen	14	10
Zhou et al. (35)	UVA	Adjuvant	31	Taxel/anthracyclin-based, CMF	Asian	Operative specimen	10	10
Schmidt et al. (38)	Survival Curve	Adjuvant/neoadjuvant	103	Taxel-based	European	NR	14	1
Park et al. (36)	UVA	Adjuvant	1551	NR	Asian	Operative specimen	50	1
Pistelli et al. (37)	UVA	Adjuvant	81	Anthracyclin-based, CMF	European	Operative specimen	30	10
Hao et al. (40)	MVA	Adjuvant	571	NR	Asian	Operative specimen	35	1
Khalifa et al. (41)	UVA	Adjuvant/neoadjuvant	106	Taxel/anthracyclin-based	European	Pre-NAC	20	10
Liu et al. (42)	UVA	Adjuvant	154	NR	Asian	Operative specimen	30	1
Asano et al. (39)	UVA	Neoadjuvant	61	Taxel/anthracyclin-based	Asian	Pre-NAC	14	1
Wang et al. (43)	MVA	Adjuvant	363	Taxel/anthracyclin-based	Asian	Operative specimen	40	1
Yang and Han (50)	UVA	Adjuvant	199	NR	Asian	Operative specimen	14	NR
Yue et al. (44)	MVA	Adjuvant	192	NR	Others	Operative specimen	50	1
Zakaria et al. (45)	MVA	Adjuvant	77	Taxel/anthracyclin-based	Others	Operative specimen	10	10
Zhong et al. (46)	UVA	Adjuvant	90	NR	Asian	Operative specimen	14	1
Ieni et al. (47)	MVA	Adjuvant	65	NR	European	Operative specimen	20	1
Kwon et al. (48)	UVA	Adjuvant/neoadjuvant	230	Taxel/anthracyclin-based	Asian	Pre-NAC	20	1
Najafi et al. (49)	Survival Curve	Adjuvant	119	NR	Asian	Operative specimen	20	10
Wang and Liu (51)	MVA	Adjuvant	110	NR	Asian	Operative specimen	20	NR

*UVA, unitivariate analysis; MVA, multivariate analysis; NR, not reported; NAC, neoadjuvant chemotherapy; CMF, cyclophosphamide, methotrexate, and 5-fluorouracil; CEF, cyclophosphamide, epirubicin, and 5-FU; EC, epirubicin/cyclophosphamide*.

### Relationship Between Ki-67 Expression and Prognosis

**Figure 2:**
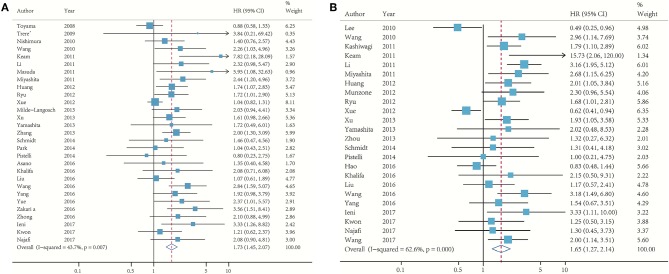
Meta-analysis of the association between Ki-67 and DFS **(A)** and OS **(B)**.

In Figure 2, 29 studies reported the association between Ki-67 and DFS, whereas 24 determined the OS. The pooled HR of DFS comparing the high Ki-67 expression level to the low was 1.73 (95% CI: 1.45–2.07; *p* < 0.001; Figure 2A). No significant heterogeneity (*I*^2^ = 43.7%) was found, and the fixed effect model was used. The pooled HR of OS was 1.65 (95% CI: 1.27–2.14; *p* < 0.001; Figure 2B), and moderate heterogeneity (*I*^2^ = 62.6%) existed among these studies.

### Subgroup Analyses

**Figure 3:**
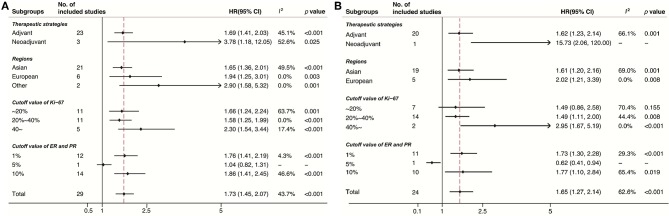
Subgroup analysis of the association between Ki-67 and DFS **(A)** and OS **(B)**.

Subgroup analyses were conducted in accordance with Ki-67 cutoffs, positive ER/PR expression thresholds (1% or 10%), treatment strategies (neo-adjuvant or adjuvant), and geographic regions (Europe, Asian, or other regions). Despite the limited number of studies in some subgroups, the results of DFS (Figure 3A) and OS (Figure 3B) stratified by these factors were consistent. Noticeably, the pooled HR for DFS and OS was 2.30 (95% CI 1.54–3.44, *p* < 0.001) and 2.95 (95% CI 1.67–5.19, *p* < 0.001), respectively, under the circumstance of a cutoff of Ki-67 staining ≥40%.

### Publication Bias

**Figure 4:**
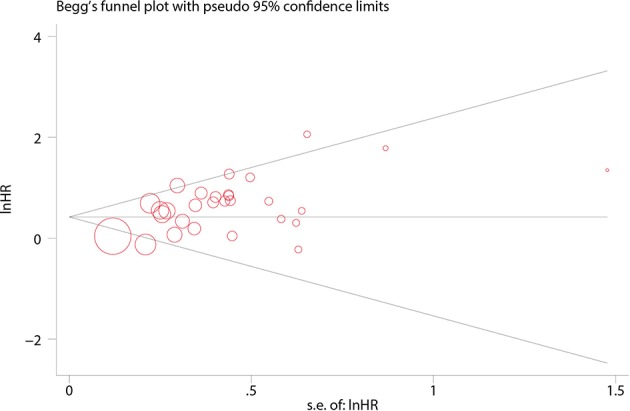
Funnel plot for the publication bias tests.

In Begg's plots of publication bias, *p*-value was 0.209 (Figure 4), implying that publication bias did not exist in the present meta-analysis.

## Discussion

TNBC has a worse prognosis than other phenotypes of breast cancer because of its aggressive biology and insensitivity to targeted therapy (52). Biomarkers useful in the selection of appropriate treatment strategies and the prediction of prognosis should be identified.

Previous studies demonstrated the prognostic role of Ki-67, as a critical biomarker of cell proliferation, in various malignancies that originate from organs and tissues, such as prostate, stomach, esophagus, cervix, and breast. A high expression level of Ki-67 protein was accompanied with poor prognostic outcomes (53). Several meta-analyses have shown that a high Ki-67 expression level is associated with the likelihood of achieving a pathological complete response (pCR) after patients with TNBC receive neo-adjuvant chemotherapy (NAC), and these patients may have favorable outcomes. Nevertheless, most of these studies included small sample sizes and contained diverse cut-offs of Ki-67 (54, 55).

In this meta-analysis, data were pooled to assess the prognostic value of Ki-67 in patients who suffered from resected TNBC and received neo-adjuvant or adjuvant chemotherapy. The results showed that patients with a high Ki-67 expression substantially had worse DFS and OS than their counterparts regardless of treatment strategies, study regions, Ki-67 cutoffs, or ER/PR thresholds.

Despite the consistency obtained in our study, the optimized cutoff of Ki-67 is still under deliberation (56). Some investigators suggested that Ki-67 should be used as a continuous marker to fully reflect the biological behavior of tumor proliferation and simultaneously resolve the cutoff issue; however, confronting diverse therapeutic strategies is impractical for clinical decision making (7). A previous meta-analysis indicated that a 25% cutoff of Ki-67 is adequate to distinguish patients with breast cancer at different risks of death (57). The cutoff selection of Ki-67 may be apparent if this parameter is considered within each subtype, and a 14% cutoff for the classification of luminal A and luminal B cancers was proposed in the 2011 St. Gallen Consensus (9). Considering that the baseline Ki-67 values of TNBC are usually higher than those of luminal diseases, Leskandarany et al. reported that the optimized Ki-67 cutoff within a TNBC subgroup population is 70% as determined by X-tile (58). Different Ki-67 values were selected as a cut-point in our included studies, and the threshold of Ki-67 varied between 10 and 50%. The subgroup analysis based on the Ki-67 cutoff indicated that the prediction was significant in all of the subgroups expect one subgroup (Ki-67 < 20%). This finding might indicate that further prospective studies should be performed to optimize the cutoff of Ki-67 in TNBC.

Baseline Ki-67 confirms the high chemosensitivity of highly proliferating TNBC after patients receive NAC, TNBC with a high Ki-67 expression likely has a high rate of pCR, which predicts favorable outcomes (59). However, studies have shown that TNBC with a high Ki-67 expression is associated with a poor prognosis because of rapid recurrence within 3 years despite a high pCR rate. A Korean study has demonstrated that a high Ki-67 expression (≥10%) is significantly associated with poor relapse-free survival and OS in preoperative TNBC despite a high pCR rate (26). Our subgroup analyses showed that a high Ki-67 expression is an adverse prognostic factor of DFS and OS both in the two groups of patients treated with adjuvant or neo-adjuvant therapy. Keam et al. reported that patients who suffer from TNBC and receive neo-adjuvant therapy with a high Ki-67 expression have a pattern of early recurrence. By contrast, the low-Ki-67-expressing subgroup did not have any pattern, indicating that a high Ki-67 expression, which indicated a high proliferation potential, might result in early recurrence. This phenomenon might partly explain why a high Ki-67 expression remained an adverse prognostic factor in the neo-adjuvant subgroup (23).

The American Society of Clinical Oncology and the College of American Pathologists Guideline Recommendations indicated that the cutoff for positive ER or PR should be ≥1% of immunoreactive tumor cell nuclei in 2010, and the previous threshold was >10%. Hence, a subgroup analysis classified by ER cut-off was performed. The results showed that a high Ki-67 expression was an adverse prognostic factor of all the subgroups, indicating that Ki-67 might be a prognostic factor of patients whose ER expression ranged from 2 to 10. Another study showed that defining triple-negative breast cancer as HER2-negative breast cancer with <10% rather than <1% of ER and progesterone receptor expression because HER2-negative primary breast cancer with ER < 10% clinically behaves like TNBC in terms of survival outcomes (60). This phenomenon might partly explain why Ki-67 was a poor prognostic factor of this patient subgroup.

Subgroup analyses on regions where these studies were conducted yielded the following classifications: Europe, Asia, and others. The results showed that a high Ki-67 expression was consistently an adverse prognostic factor of DFS and OS in these three subgroups. Moreover, the pooled data showed that TNBC was more likely to recur in Europe than in Asia. However, only eight studies were from Europe, while 27 studies were from Asia. Therefore, these findings should be carefully considered, and further studies should be performed to verify these results.

Notably, our study has a few limitations. First, due to linguistic constraints, we included studies written in English and Chinese only, hence publications in other languages could have been omitted. Second, we failed to perform subgroup analyses on other parameters, such as age or tumor stage, because of insufficient background information and thus might cause heterogeneity in the pooled results. Other clinical heterogeneities among studies, such as different NAC and adjuvant regimens, were not analyzed.

In conclusion, this study demonstrated that higher Ki-67 expression is a poorer prognostic factor of resected TNBC. The cut-off of ki-67 ≥40% is associated with a greater risk of recurrence and death compared with lower expression rates, despite the Ki-67 threshold with the greatest prognostic significance is as yet unknown.

## Data Availability Statement

All datasets generated for this study are included in the manuscript/supplementary files.

## Author Contributions

QW, WLi, and QZ contributed to the conception and design of this research. QW, GM, YD, WLu, YZ, WLi, and QZ contributed to the drafting of the article and final approval of the submitted version, and contributed to data analyses and the interpretation and completion of the figures and tables. All authors read and approved the final manuscript.

### Conflict of Interest

The authors declare that the research was conducted in the absence of any commercial or financial relationships that could be construed as a potential conflict of interest.
